# Lipidomics random forest algorithm of seminal plasma is a promising method for enhancing the diagnosis of necrozoospermia

**DOI:** 10.1007/s11306-024-02118-x

**Published:** 2024-05-21

**Authors:** Tianqin Deng, Wanxue Wang, Zhihong Fu, Yuli Xie, Yonghong Zhou, Jiangbo Pu, Kexin Chen, Bing Yao, Xuemei Li, Jilong Yao

**Affiliations:** 1 Reproductive Medicine Centre, Shenzhen Maternity & Child Healthcare Hospital, Fuqiang Road No.3012, Shenzhen, 51807 China; 2 Newborn Screening Centre, Shenzhen Maternity & Child Healthcare Hospital, Shenzhen, China; 3 Reproductive Medicine Centre, Nanjing Jinling Hospital, The First School of Clinical Medicine, Southern Medical University (General Hospital of Eastern Military Region), Nanjing, China

**Keywords:** Lipidomics, Necrozoospermia, Seminal plasma, Random forest, Liquid chromatography–mass spectrometry (LC–MS)

## Abstract

**Background:**

Despite the clear clinical diagnostic criteria for necrozoospermia in andrology, the fundamental mechanisms underlying it remain elusive. This study aims to profile the lipid composition in seminal plasma systematically and to ascertain the potential of lipid biomarkers in the accurate diagnosis of necrozoospermia. It also evaluates the efficacy of a lipidomics-based random forest algorithm model in identifying necrozoospermia.

**Methods:**

Seminal plasma samples were collected from patients diagnosed with necrozoospermia (n = 28) and normozoospermia (n = 28). Liquid chromatography–mass spectrometry (LC–MS) was used to perform lipidomic analysis and identify the underlying biomarkers. A lipid functional enrichment analysis was conducted using the LION lipid ontology database. The top 100 differentially significant lipids were subjected to lipid biomarker examination through random forest machine learning model.

**Results:**

Lipidomic analysis identified 46 lipid classes comprising 1267 lipid metabolites in seminal plasma. The top five enriched lipid functions as follows: fatty acid (FA) with ≤ 18 carbons, FA with 16–18 carbons, monounsaturated FA, FA with 18 carbons, and FA with 16 carbons. The top 100 differentially significant lipids were subjected to machine learning analysis and identified 20 feature lipids. The random forest model identified lipids with an area under the curve > 0.8, including LPE(20:4) and TG(4:0_14:1_16:0).

**Conclusions:**

LPE(20:4) and TG(4:0_14:1_16:0), were identified as differential lipids for necrozoospermia. Seminal plasma lipidomic analysis could provide valuable biochemical information for the diagnosis of necrozoospermia, and its combination with conventional sperm analysis may improve the accuracy and reliability of the diagnosis.

**Supplementary Information:**

The online version contains supplementary material available at 10.1007/s11306-024-02118-x.

## Background

Necrozoospermia was defined as the presence of less than 58% of alive spermatozoa in semen according to the sixth edition of World Health Organization manual, the test to assess sperm vitality is Eosin-nigrosin staining (WHO, 2021). Necrozoospermia, with an incidence of approximately 0.2–0.5% in male infertility (Boursier et al., 2022). The causes of necrozoospermia are multiple: genital tract infections, testicular hyperthermia, varicocele, hyperthyroidism, spinal cord injury, polycystic kidney disease, anti-sperm antibodies, advanced male age, toxic substances, In 20% of total cases, the cause of necrozoospermia is unknown (Agarwal et al., 2022). There was a relatively limited approach to the management of necrozoospermia, for example: avoiding and treating underlying risks and conditions, frequent ejaculation, drug. However, the treatment effect is not good, and the majority of patients finally obtain pregnancy through assisted reproductive technology, and the sperm was which the use of ICSI with ejaculated or surgically extracted spermatozoa. Magnetic-activated cell sorting was a novel methods for the elimination of dead spermatozoa (Máté et al., 2022). Our previous preliminary research suggests that the occurrence of necrozoospermia is associated with choline and the arachidonic acid metabolic pathway (Deng et al., 2023).

Seminal plasma contains rich substance components, including sugars, lipids, proteins, and trace elements. These components have the potential to influence the occurrence and development of spermatozoa, while also objectively reflecting the functional status of it (Boguenet et al., 2020). Lipids in seminal plasma include cholesterol (free and esterified cholesterol), TGs, phospholipids (SM, PC, and PE), and prostaglandins of the E series. A previous study indicated that alterations in lipid composition in seminal plasma can impact the fertility of spermatozoa, potentially leading to the loss of fertilization function and causing male infertility (Kawano et al., 2011). Therefore, research on the lipid composition of seminal plasma holds significant importance. Lipids play a crucial role in spermatozoa maturation, energy acquisition, and fertilization processes (Kawano et al., 2011). Seminal plasma, as a non-invasive sample, is particularly suitable for discovering the causes of unexplained male infertility and conducting mechanism studies.

Lipidomics, a high-throughput analytical technique based on liquid chromatography–mass spectrometry (LC–MS), systematically analyzes the lipid composition and expression patterns in biological systems (Dalli et al., 2018). Lipidomic analysis efficiently investigates changes in lipid families and compounds during various biological processes, elucidating relevant biological mechanisms and principles.

This study aims to systematically profile the lipid composition in seminal plasma and to ascertain the potential of lipid biomarkers in the accurate diagnosis of necrozoospermia. It also evaluates the efficacy of a lipidomics-based random forest algorithm model in identifying necrozoospermia.

## Materials and methods

### Semen sample collection and analysis

The study included healthy donors and infertility patients from the Reproductive Medicine Center at Shenzhen Maternity & Child Healthcare Hospital (SZMCH) and approved by the ethics committee (No. SFYLS [2021]038). There are 28 participants in the control group, volunteers had at least two normal semen analysis results, meeting laboratory manual for the examination and processing of human semen (WHO 6th edition) (WHO, 2021). Volunteers in control group were healthy, free from genetic diseases, and sought consultation owing to female-related infertility factors. The case group comprised 28 patients who have been diagnosed as necrozoospermia, with normal hormone levels and no other abnormal physical conditions. Patients were included in the necrozoospermia group if they met the following criteria: spermatozoa forward motility percentage of 0%, spermatozoa viability (eosin–nigrosin staining) < 58%, and sperm concentration equal to or higher than the lower limit of the reference range.

The exclusion criteria were as follows: obesity; metabolic diseases, such as diabetes and thyroid dysfunction; recent acute urinary tract infections, chlamydia, or mycoplasma infections; leukocytospermia; exposure to substances toxic to the reproductive system; abstinence time > 7 days or < 2 days; semen volume < 1 mL.

Computer-assisted semen analysis (CASA) system has been used to analyze the volume, concentration, and motility. Eosin–nigrosin staining was employed to assess spermatozoa viability, and Diff–Quik staining was used for spermatozoa morphology analysis. The remaining sperm was centrifuged at 3000×*g* for 15 min, and seminal plasma samples were stored at − 80 °C for subsequent lipidomic analysis.

### Lipid extraction

Chloroform–methanol solution (2:1, v/v) was added to the sample at − 20 °C. After a series of repeated vortexing and centrifugation for purification, the sample was concentrated using a vacuum concentrator. Finally, the sample was dissolved in isopropanol, filtered through a 0.22 μm membrane, resulting in the test sample for subsequent LC–MS steps.

### LC–MS analysis

The instrument utilized an electrospray ionization source (ESI) operating in both positive and negative ionization modes. The positive ion spray voltage was set at 3.50 kV, and the negative ion spray voltage was set at 2.50 kV. The sheath gas was maintained at 30 arb, and the auxiliary gas at 10 arb. The capillary temperature was set to 325 °C, and a resolution of 35,000 was employed for full scan analysis within the range of 150 to 2000. Higher-energy collisional dissociation (HCD) was used for second-stage fragmentation with a collision voltage of 30 eV. Dynamic exclusion was implemented to eliminate unnecessary MS/MS information (Dalli et al., 2018).

### Data processing

The raw data obtained from MS were individually processed using LipidSearch software (V4.2.28; Thermo Fischer Scientific). This involved lipid annotation, peak alignment, and peak filtering. The key parameters included retention time (R.T.) tolerance = 0.25, m-score threshold = 3. The outcome was a quantitative list containing information, such as lipids, mass-to-charge ratio (m/z), retention time, and peak intensity. To facilitate comparison among data of different magnitudes, sum peak normalization was performed to correct quantitative values.

### Statistical and bioinformatical analysis

Owing to the multidimensional nature of lipidomic data and the high correlation between certain variables, traditional univariate analysis falls short in rapidly, comprehensively, and accurately mining potential information within the data. Therefore, before conducting multivariate statistical analysis on lipidomic data, it is necessary to appropriately transform the data through weighting, i.e., scaling. In this analysis, a self-weighted approach was applied to the data prior to sample classification to obtain more reliable and intuitive results. The multivariate statistical analysis methods employed in this analysis, utilizing the R package Ropls, include (1) principal component analysis (PCA); (2) partial least squares-discriminant analysis (PLS-DA); and (3) orthogonal PLS-DA (OPLS-DA).

The Statistical Package for Social Sciences for Windows (SPSS 26, IBM Corp., Armonk, NY) was used for statistical analyses. A p-value < 0.05 was considered statistically significant. The distribution of data was evaluated by using the Kolmogorov–Smirnov test. Student's *t*-test or the Mann–Whitney Wilcoxon test was used for comparisons.

For correlation analysis, correlation coefficients tending towards 1 or − 1 indicate a strong linear relationship. A positive correlation tends towards 1, and a negative correlation towards − 1. Statistical testing of lipid association analysis was conducted using the cor.test() function in R, with a p value of less than 0.05 indicating significant correlation (Kawano et al., 2011).

The R package lipidomeR was employed for carbon chain length and carbon saturation analysis of lipids. Correlation network analysis was conducted for differential lipids, and the Leiden community classification algorithm was used for community classification of associated differential lipids. For lipids classified into different communities, the R package for LION software was used for functional enrichment analysis. Lipid functional enrichment analysis utilized the LION lipid ontology database (Molenaar et al., 2019), which links over 50,000 lipid species to four major branches: (1) lipid classification (LIPIDMAPS classification hierarchy); (2) chemical and physical properties (e.g., fatty acid [FA] length and unsaturation, functional group charge, and membrane fluidity); (3) function; and (4) subcellular components (major subcellular localization).

## Results

### Semen analysis

There were no statistically significant differences in age and abstinence time between the two groups (p > 0.05). However, there were statistically significant differences between the two groups in semen volume, pH value, spermatozoa concentration, forward motility percentage, percentage of normal spermatozoa morphology, survival rate, seminal plasma neutral α-glucosidase, and seminal plasma zinc levels (p < 0.05) (Table 1).

**Table 1:** Semen analysis results

Parameter	Experimental group (n = 28)	Control group (n = 28)
Age (years)	36.5 ± 7.59	36.8 ± 7.04*
Abstinence days	4.32 ± 1.19	4.36 ± 1.03*
Semen volume (mL)	1.91 ± 1.02	3.18 ± 1.39**
pH	6.98 ± 0.36	7.28 ± 0.16**
Sperm concentration (106 per mL)	66.83 ± 101.37	80.79 ± 44.56**
Forward motility (%)	0.00 ± 0.00	44.24 ± 6.34**
Sperm survival rate (%)	21.71 ± 14.57	74.03 ± 8.34**
Seminal plasma α-glucosidase (µmol/ejaculation)	30.41 ± 22.52	60.1 ± 36.17**
Seminal plasma zinc (µmol/ejaculation)	5.72 ± 3.30	8.15 ± 4.64**

*p > 0.05; **p < 0.05

### Quality control of LC–MS

**Fig. 1:**
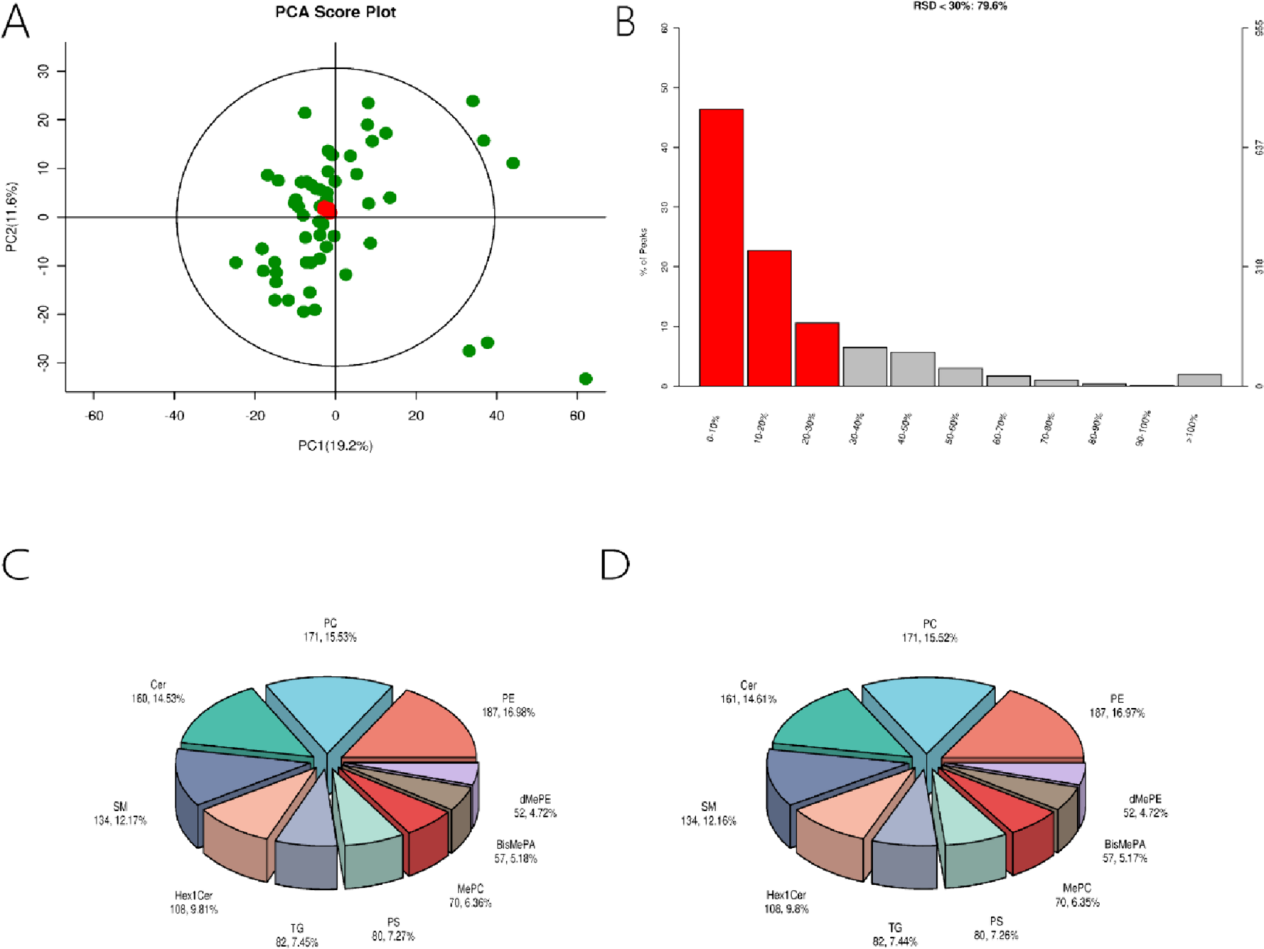
LC–MS sample quality control. **A** PCA score plot: x-axis shows the first principal component, y-axis the second; red dots are QC samples showing good reproducibility within 95% confidence interval, green dots are non-QC samples. **B** RSD distribution plot: left axis shows proportion, right axis shows quantity, horizontal axis shows RSD range. **C** Control group lipid classification pie chart. **D** Necrozoospermia group lipid classification pie chart. *PCA* principal component analysis; *RSD* relative standard deviation; *BisMePA* bis-methylphosphatidic acid; *Cer* ceramide; *dMePE* dimethylphosphatidylethanolamine; *Hex1Cer* monohexosylceramide; *MePC* methylphosphatidylcholine; *PC* phosphatidylcholine; *PE* phosphatidylethanolamine; *PS* phosphatidylserine; *SM* sphingomyelin; *TG* triglyceride (Color figure online)

The PCA score plots (Fig. 1A) show minimal variation in QC samples, indicating high method stability and data quality, with good reproducibility evidenced by clustering within the 95% confidence interval. About 79.6% of feature peaks in QC samples had an RSD < 30%, ensuring high-quality data for biomarker detection (Fig. 1B). Lipids were categorized into groups. In the control group, 53 lipid categories with 1591 species were identified, and the top 10 are shown in a pie chart (Fig. 1C). Similarly, the experimental group had 53 categories with 1592 lipid species, with the top 10 displayed in a pie chart (Fig. 1D).

The Supplementary Datasheet 1 provides a comprehensive tabulation of various ceramide species, including their identification and quantification details. This dataset enumerates the different types of Cer, AcCa, and BisMePA molecules, detailing their mass-to-charge ratios (m/z), retention times (rt), and the relative abundance of their respective ions across multiple samples. It is crucial for understanding the quantitative analysis of these lipid species within the context of the study.

### PCA, OPLS-DA

**Fig. 2:**
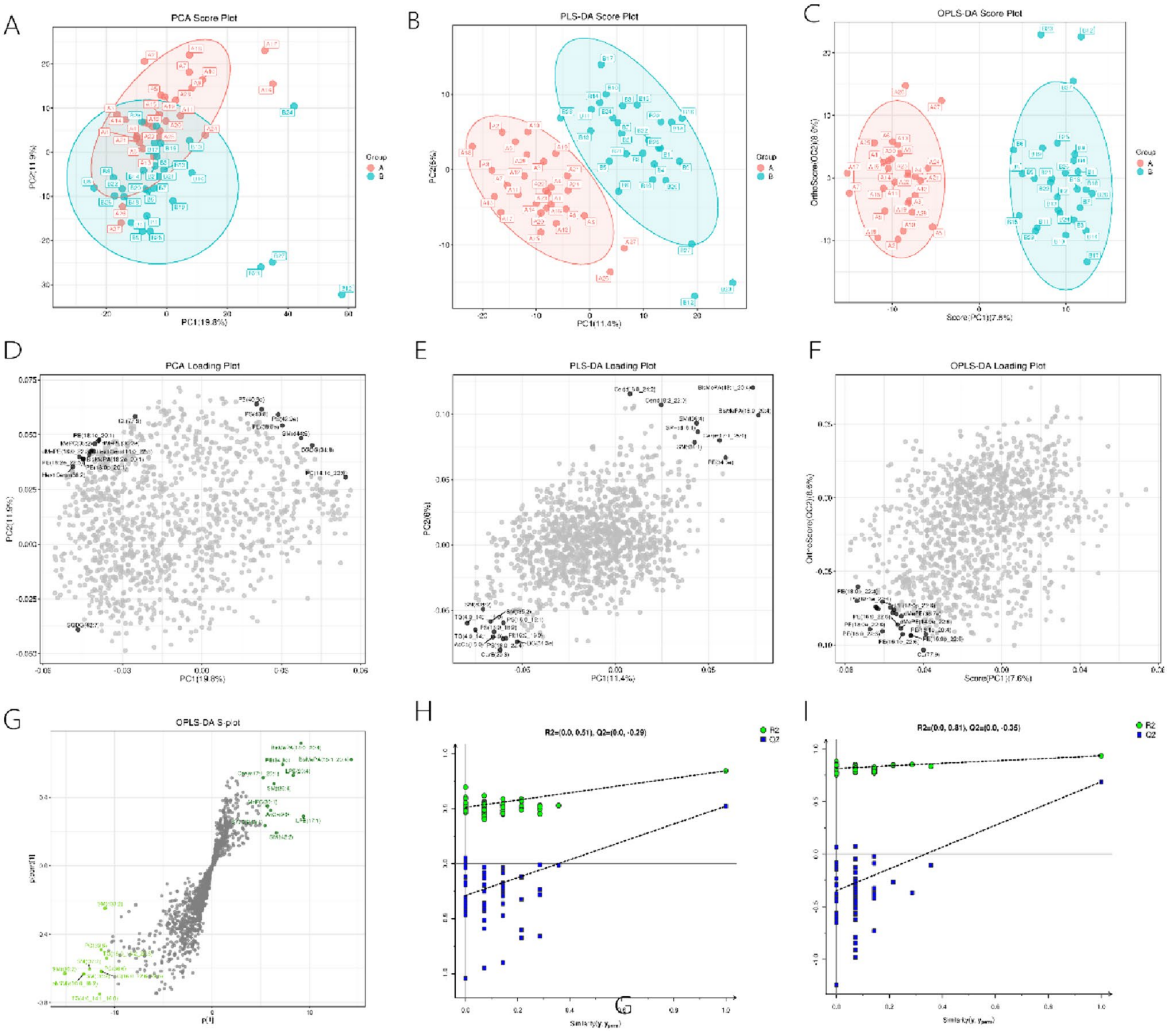
Presents the results of multivariate statistical analysis for dimension reduction and classification. **A** PCA score plot demonstrates groupings, with the x and y-axes showing the interpretation degree of the first and second principal components respectively; blue dots represent the necrozoospermia group, while pink dots denote the control group. **B** PLS-DA Score plot and **C** OPLS-DA Score plot follow a similar format, further distinguishing between groups. **D** PCA, **E** PLS-DA, and **F** OPLS-DA Loading plots depict the relationship between metabolic molecules and principal components. **G** OPLS-DA S-plot highlights lipids correlated with key biological components, with significant lipids marked in the upper right and lower left corners. **H** PLS-DA and **I** OPLS-DA Permutation test plots assess the models’ reliability, with effective models indicated by lower blue Q2 points than the rightmost blue Q2, and regression lines intersecting the y-axis below 0. *PCA* principal component analysis; *PLS-DA* partial least square discriminant analysis; *OPLS-DA* orthogonal partial least square discriminant analysis (Color figure online)

The PCA model, characterized by an R2X(cum) value of 0.529, is substantiated by the tightly clustered distribution of both sample and quality control (QC) data points on the PCA score plot (Fig. 2A). This pattern of distribution suggests that the metabolic profiles among the samples are consistent, which is indicative of high-quality data acquisition where the analytical variance is less than the biological variance.

OPLS-DA, an advanced version of PLS-DA, provided a more refined analysis by reducing model complexity and honing in on relevant information from the X matrix. The OPLS-DA model, with improved validation parameters [R2X(cum) of 0.416, R2Y(cum) of 0.934, and Q2(cum) of 0.686], offered enhanced interpretability and predictability (Figs. 2B, D, F, G). The OPLS-DA S-plot, in particular, was instrumental in identifying lipids strongly correlated with major components in biological processes. The distinct representation of the necrozoospermia and control groups, depicted as blue and pink dots respectively, underscored the effectiveness of these methods in distinguishing metabolic variations between groups. This comprehensive approach to metabolomic analysis highlighted the significance of multivariate statistical methods in revealing intricate details of metabolic differences.

### Differential analysis

**Fig. 3:**
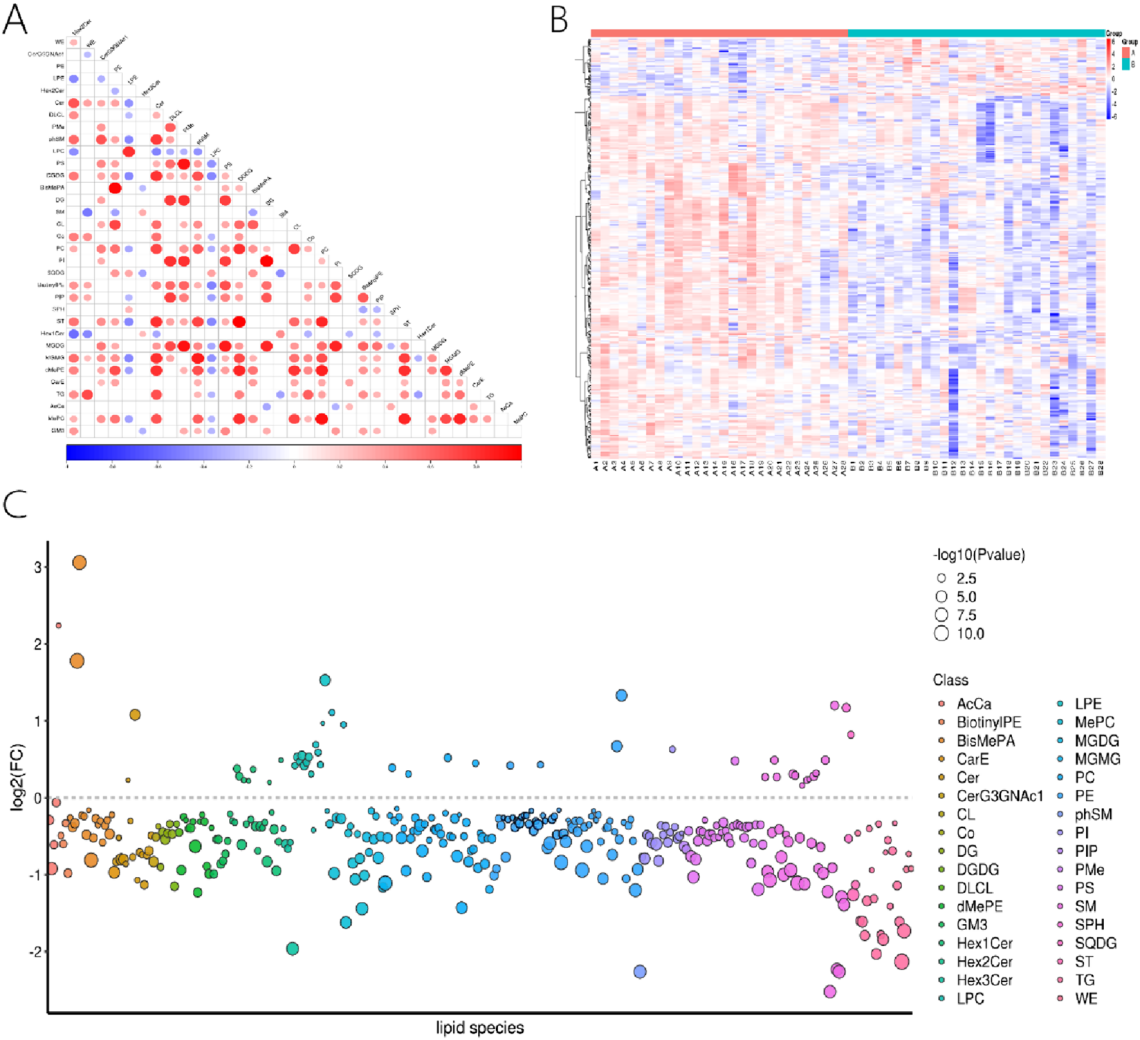
Analysis of differential lipid compounds. **A** The correlation heat map shows the correlation between different lipid classes, with red dots indicating positive correlation and blue dots negative correlation. The color intensity reflects the strength of the correlation, and dot size indicates the significance of the correlation (p value). **B** The clustering heat map displays quantitative lipid values, with color intensity indicating expression levels. It includes a lipid clustering tree and a sample clustering tree. **C** The lipid class bubble chart plots the log2 fold change of lipids against their classes, using dot color to differentiate lipid categories and dot size to represent the significance of the differences (p value) (Color figure online)

There are 373 lipid compounds with statistical differences identified using preset p-value thresholds and variable importance for the projection in the OPLS-DA model (VIP). These included 51 upregulated and 322 downregulated lipid species (see Table 2, Fig. 3).

**Table 2:** Differential lipid results (partial)

Name	m/z	p-value	VIP	log2(FC)	IonFormula	Regulation
TG (4:0_14:1_16:0)	626.5354155	4.52E−11	2.72	− 2.13	C37 H72 O6 N1	Down
BisMePA (18:0_20:4)	770.5694335	4.06E−10	2.59	1.78	C43 H81 O8 N1 P1	Up
MGMG(32:1)	773.5784385	1.64E−09	2.70	− 1.11	C43 H81 O11	Down
PE(18:1e_22:4)	778.5756155	2.21E−09	2.60	− 0.94	C45 H81 O7 N1 P1	Down
SM(d36:2)	773.5814295	2.25E−09	2.70	− 1.11	C42 H82 O8 N2 P1	Down
PE(18:0p_22:4)	780.5901685	2.27E−09	2.58	− 0.84	C45 H83 O7 N1 P1	Down
BisMePA (18:2e_22:4)	780.5901685	2.70E−09	2.62	− 0.81	C45 H83 O7 N1 P1	Down
BisMePA (18:1_20:4)	768.5537835	3.56E−09	2.25	3.06	C43 H79 O8 N1 P1	Up
TG (4:0_14:1_18:0)	654.5667155	3.64E−09	2.50	− 1.73	C39 H76 O6 N1	Down
SM(d18:1_18:1)	729.5905025	4.63E−09	2.63	− 1.07	C41 H82 O6 N2 P1	Down

*m/z* mass-to-charge ratio; *VIP* variable importance for the projection in the OPLS-DA model; *log2(FC)* logarithmic fold change, indicating the magnitude of change; *IonFormula* molecular formula; *Regulation* direction of regulation (up or down)

The data was utilized to create a cluster heat map of different lipid classes. Furthermore, the correlation between various lipid classes was analyzed by calculating the Pearson correlation coefficients among them. Lipids changing in the same direction showed positive correlation, while those changing in opposite directions displayed negative correlation.

For each comparison group, differential lipids were visualized using a lipid bubble chart plotted with P values and FC values. Different lipid classes were represented with distinct colors, with each bubble representing a single lipid. This bubble chart intuitively displayed the expression synchrony and differences among various classifications.

### Correlation analysis

The correlations between discriminative lipids and seminal plasma analysis parameters were assessed using Spearman’s rank correlation coefficients. Table 3 displays the correlation coefficients, two-tailed p-values, and the statistical significance of these correlations. The results indicated that age did not show significant correlations with lipid classes or seminal plasma parameters, as all p-values were above 0.05, suggesting a limited impact of age on these parameters.

**Table 3:** Correlation of discriminative lipids and lipid classes with semen analysis parameters

	Choline		Betaine		TMAO		Creatinine		Carnitine	
	Correlation coefficient	p-value (two-tailed)	Correlation coefficient	p-value (two-tailed)	Correlation coefficient	p-value (two-tailed)	Correlation coefficient	p-value (two-tailed)	Correlation coefficient	p-value (two-tailed)
Age	− 0.158	0.342	0.136	0.415	0.251	0.129	0.093	0.579	− 0.088	0.599
Abstinence days	− 0.214	0.197	0.172	0.302	0.106	0.526	0.042	0.801	0.138	0.409
Semen volume	− 0.159	0.341	0.265	0.108	0.056	0.739	− 0.065	0.698	0.581**	0
pH	− 0.163	0.328	− 0.05	0.766	− 0.372*	0.021	− 0.282	0.087	0.564**	0
Sperm concentration	− 0.29	0.078	0.201	0.225	0.11	0.51	− 0.099	0.553	0.585**	0
Progressive Motility	− 0.209	0.207	0.311	0.058	− 0.193	0.245	− 0.204	0.218	0.825**	0
Normal Sperm Morphology Rate	− 0.124	0.457	0.192	0.248	0.019	0.911	− 0.054	0.747	0.329*	0.044
Hypoosmotic Swelling Test	− 0.094	0.582	0.093	0.584	− 0.274	0.1	− 0.215	0.202	0.736**	0
Polymorphonuclear Neutrophil Elastase	− 0.125	0.454	− 0.154	0.355	− 0.134	0.423	− 0.259	0.116	0.062	0.712
Neutral Alpha-Glucosidase	− 0.285	0.082	0.394*	0.014	− 0.083	0.622	− 0.344*	0.034	0.530**	0.001
Seminal plasma zinc	− 0.21	0.206	0.209	0.208	− 0.073	0.662	− 0.122	0.465	0.324*	0.047

*At the 0.05 level (two-tailed), the correlation is significant

**At the 0.01 level (two-tailed), the correlation is highly significant

A moderate positive correlation was observed between the number of abstinence days and seminal plasma volume and pH, with p-values of 0.198 and 0.302, respectively. This suggests that an increase in abstinence days may be associated with a decrease in seminal plasma volume and a lower pH value. However, the correlation between the number of abstinence days and the percentage of progressive motility and sperm viability was not significant, with p-values of 0.197 and 0.172, respectively.

Significant negative correlations were found between seminal plasma volume and certain lipid parameters, particularly with PE(18:1e_22:4) and PE(18:0p_22:4), with correlation coefficients of − 0.581 and − 0.585, respectively, and p-values less than 0.01. This indicates that a decrease in seminal plasma volume is closely associated with reduced levels of these lipid classes. Additionally, seminal plasma volume showed significant correlations with sperm concentration and progressive motility, with p-values of 0.078 and 0.207, respectively, which may imply that a decrease in seminal plasma volume is related to a decline in sperm quality and motility.

The correlations between pH value and lipid parameters were inconsistent, with significant negative correlations for PE(18:1e_22:4) and PE(18:0p_22:4), with correlation coefficients of − 0.372 and − 0.282, respectively, and p-values less than 0.05. This suggests that a decrease in pH value may be related to reduced levels of these lipid classes. However, correlations with other lipid parameters were not significant.

Sperm concentration showed significant correlations with PE(18:1e_22:4) and PE(18:0p_22:4), with correlation coefficients of − 0.585 and − 0.58, respectively, and p-values less than 0.01, indicating that a decrease in sperm concentration may be associated with reduced levels of these lipid classes. In contrast, sperm viability did not show significant correlations with lipid parameters, as all p-values were above 0.05.

### Lipid community analysis, correlation and structural feature heatmap

**Fig. 4:**
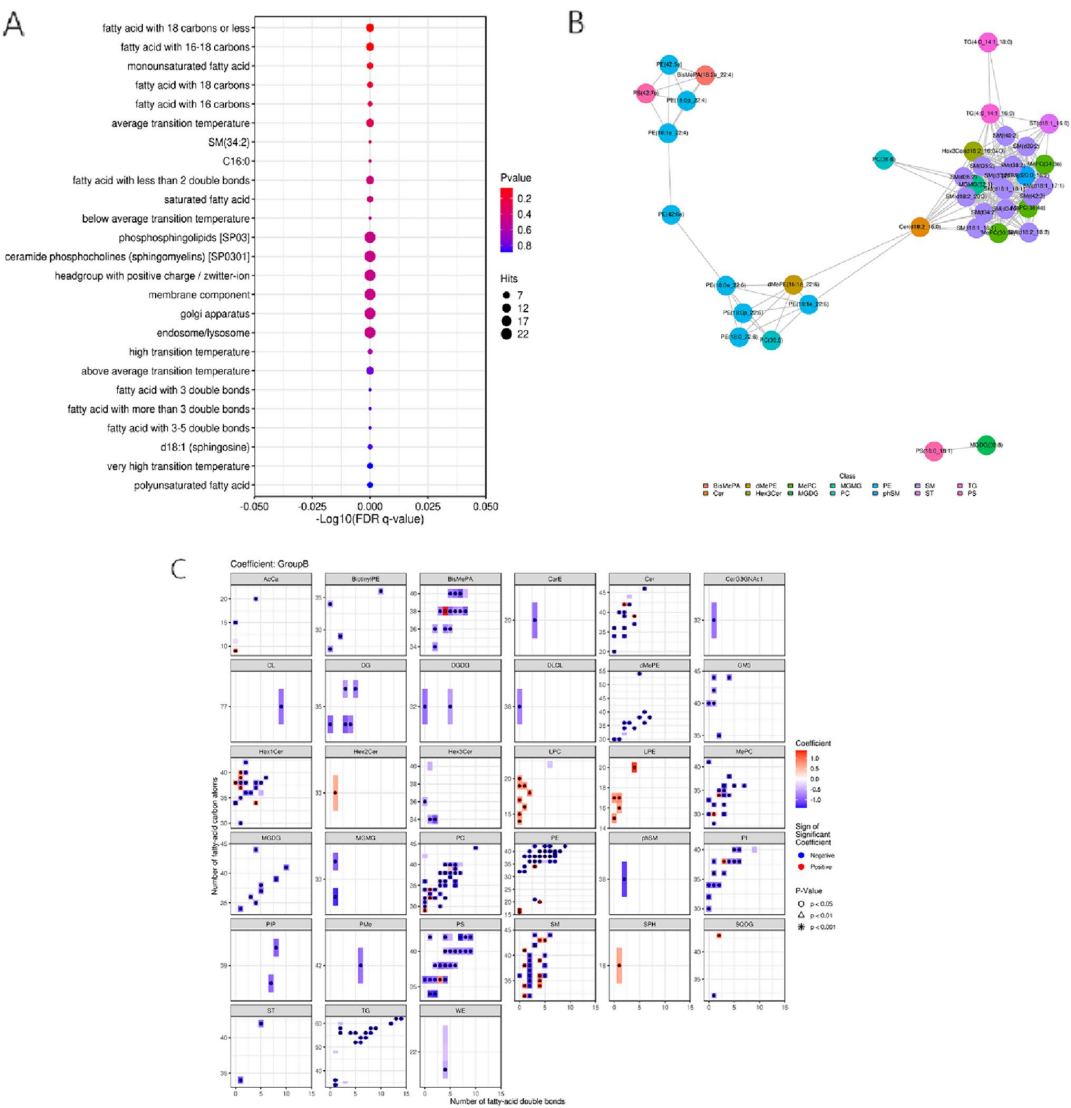
Lipidomic analysis: functional enrichment and network features. **A** LION enrichment bubble plot: Horizontally shows the − log10 FDR q-value, and vertically lists enriched LION database entries. Bubble color and size represent the enrichment p-value significance and the number of lipids enriched, respectively. **B** Interaction map of lipid molecules: Displays top 50 differential lipids in the necrospermia group with high correlation coefficients and significant p values. **C** Bee swarm plot of lipid structural differences: Dots represent lipid molecules, color-coded by Leiden community classification, with lines showing significant correlations between lipids. *FDR* false discovery rate (Color figure online)

Lipid functional enrichment analysis was conducted using the LION lipid ontology database, which encompasses over 50,000 lipid species. This analysis was focused on four key categories: lipid function, chemical and physical properties, LIPIDMAPS classification, and subcellular localization. The lipids differentiated by the Leiden community classification were the primary focus in our network analysis (Fig. 4A).

For the correlation network analysis, the top 50 lipids, showing significant p value differences, were selected based on criteria of a correlation coefficient greater than 0.8 and a p value less than 0.05. These criteria were also applied to the Leiden community classification, which included all differentially classified lipids (Fig. 4B).

Additionally, a structural feature analysis of the differential lipids was performed using the lipidomeR tool. Regression models were fitted to the lipid quantitation results to systematically interpret lipidomic characteristics. Heat maps were generated to illustrate the relationships between comparison groups and lipids, categorized by lipid class. This analysis, which computed the number of carbon atoms and saturation levels (number of double bonds) in lipids, was limited to those annotated with chain length and saturation information and focused on comparing structural feature differences between two groups (Fig. 4C).

### Machine-learning biomarker discovery

**Fig. 5:**
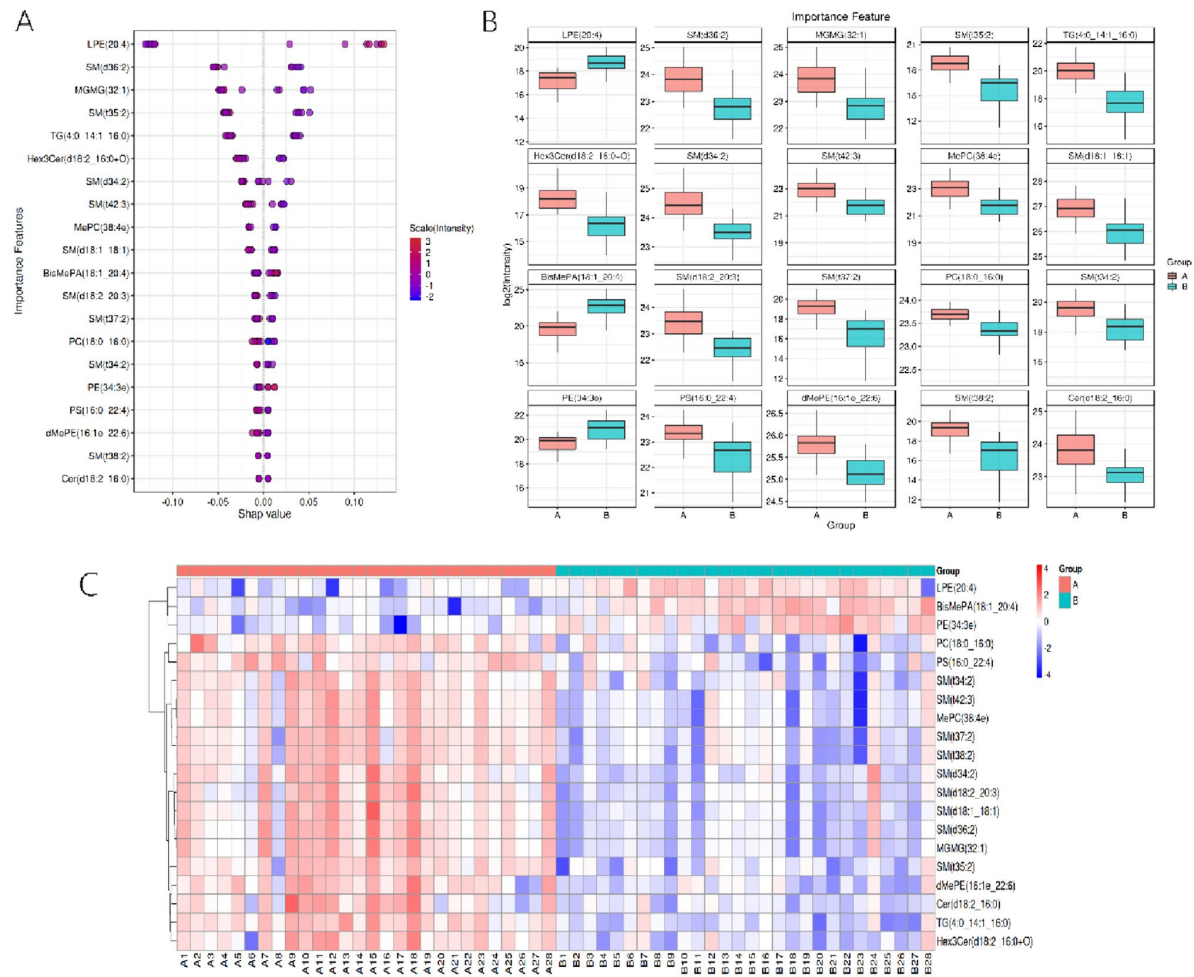
Random forest feature ranking using wrapper method. **A** Importance feature bee plot: displays the top 20 lipid features by importance on the vertical axis, with their absolute average Shapley scores on the horizontal axis, indicating lipid significance. **B** Box Plot of Important Lipid Features: Compares log2 values of substance measurements in control (a) and experimental (b) groups. Illustrates distribution changes in content. **C** Cluster Heatmap: Shows relative lipid content levels with color coding (red for higher, blue for lower). The horizontal axis lists samples, the vertical axis lists lipids, and a clustering tree is on the left (Color figure online)

The limitations of traditional statistical methods in biomarker discovery were addressed. These limitations included weak insights into feature interactions and poor predictive classification for new samples. Machine learning approaches were employed for feature selection and extraction. Methods such as PCA were utilized for feature extraction, while filter, wrapper, and embedded methods were used for feature selection. Notably, the wrapper method using random forests (RF) was applied to rank feature importance. Patterns of metabolite expression with functional correlations were revealed, visualized in a two-way hierarchical cluster heatmap (Fig. 5A). This approach identified important feature molecules based on their expression patterns.

For the analysis of feature importance, SHapley Additive exPlanations (SHAP) were used. This method offers local and global interpretability by assigning Shapley values to each feature, indicating their contribution to model predictions. SHAP was found to outperform traditional methods like RF feature importance, providing better consistency and insight into the positive or negative influence of predictors on the target variable. The absolute average of Shapley values across all samples was calculated to determine the global importance of each feature.

The top 20 lipid features identified as most important included LPE(20:4), SM(d36:2), MGMG(32:1), among others (Fig. 5B, C). These features were crucial in the context of biomarker discovery, showcasing the effectiveness of machine learning in overcoming the limitations of traditional statistical methods.

### Machine-learning ROC prediction analysis

**Fig. 6:**
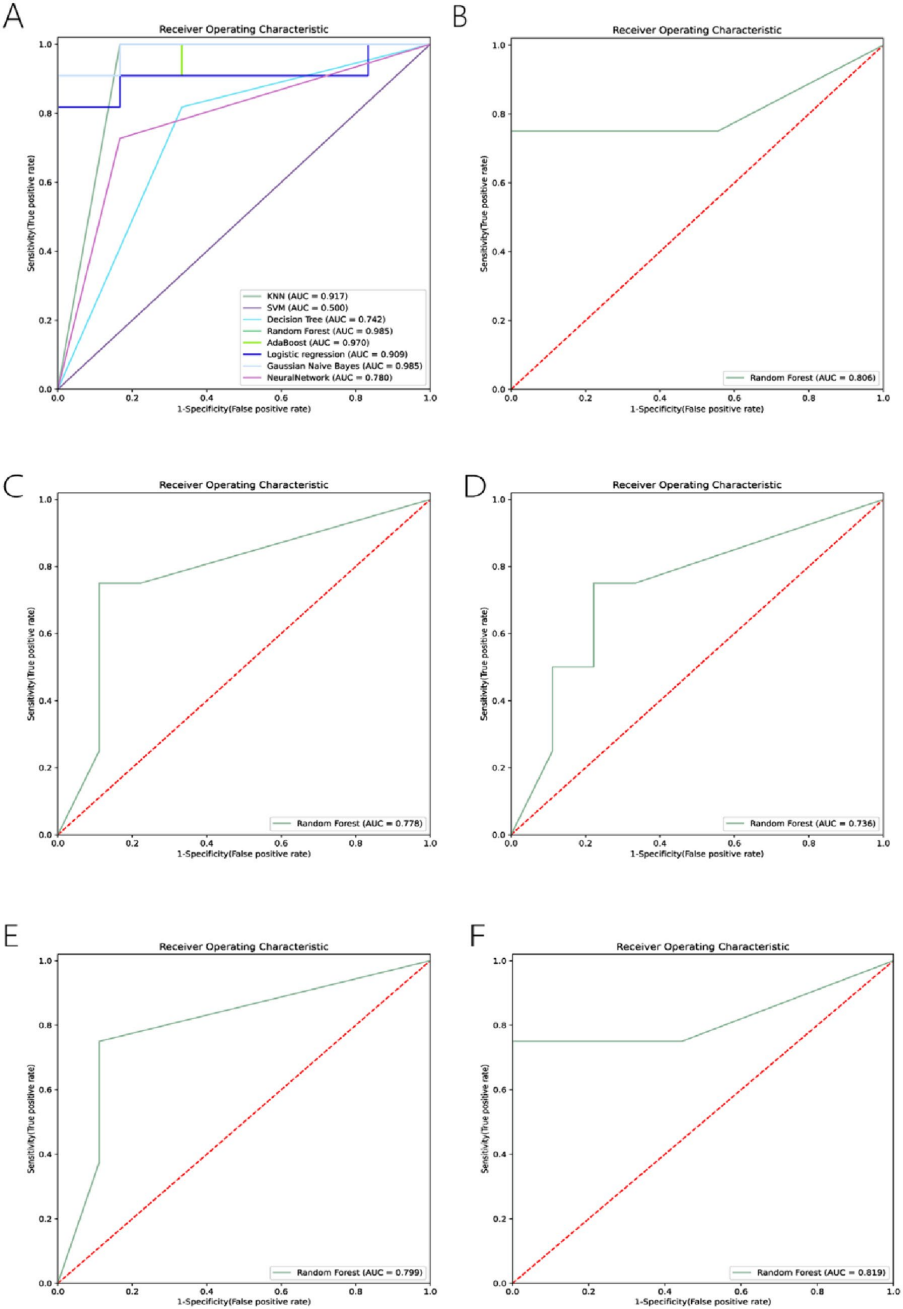
ROC prediction analysis using machine learning for top 5 biomarkers. **A** Overall machine learning ROC curve, illustrating diagnostic accuracy with lower false positive rates indicated by closer proximity to the upper-left corner. **B**–**F** Individual ROC curves for LPE(20:4), SM(d36:2), MGMG(32:1), SM(t35:2), and TG(4:0_14:1_16:0), respectively. All curves demonstrate diagnostic performance, with lower false positive rates on the horizontal axis and higher true positive rates on the vertical axis, assessing diagnostic accuracy. *KNN* K-nearest neighbor, *RF* random forest, *SVM* support vector machine, *GNB* Gaussian Navie bayes, *LR* logistic regression, *DT* decision tree, *AdaBoost* adaptive boosting; *LPE* lysophosphatidylethanolamine; *ROC* receiver operating characteristics; *TG* triglyceride

Eight models combining the above-mentioned important features were compared using ROC curves, accuracy, sensitivity, and specificity as evaluation metrics. The top five lipid features by contribution ranking were selected for RF machine learning model analysis, which include LPE(20:4), SM(d36:2), MGMG(32:1), SM(t35:2), and TG(4:0_14:1_16:0). ROC curves were plotted, and lipids with an AUC > 0.8 were identified: LPE(20:4) and TG(4:0_14:1_16:0) (Table 4, Fig. 6, also see Supplementary Datasheet 2). The results of a random forest model analysis focusing on the AUC-related parameters for two lipids, Lysophosphatidylethanolamine (LPE) with the chain length 20:4 and Triglyceride (TG) with the chain lengths 4:0, 14:1, and 16:0. This analysis includes the area under the curve (AUC), sensitivity, specificity, and their 95% Confidence Intervals (CIs). The AUC for LPE(20:4) is reported as 0.81, with a sensitivity of 0.75 and specificity of 0.56, and the respective 95% CIs for sensitivity and specificity are 0.45–1 and 0.23–0.88. In the case of TG(4:0_14:1_16:0), the AUC is slightly higher at 0.82, with a sensitivity of 0.63 and a specificity of 1.0. The 95% CIs for sensitivity range from 0.29 to 0.969, while the specificity is consistently at 1.0 across its CI. They have high AUC values and a balance between sensitivity and specificity in differentiating conditions, indicating their potential to be significant biomarkers​​.

**Table 4:** Random forest model analysis AUC-related parameters for the two featured lipids

Lipid name	AUC	Sensitivity	95% CI sensitivity	Specificity	95% CI specificity
LPE(20:4)	0.81	0.75	0.45–1	0.56	0.23–0.88
TG(4:0_14:1_16:0)	0.82	0.63	0.29–0.969	1.0	1.0–1.0

*AUC* area under the ROC curve; *CI* confidence interval; *LPE* lysophosphatidylethanolamine; *TG* triglyceride

## Discussion

Lipids are biologically organic molecules that are insoluble in water but soluble in nonpolar solvents. They can be categorized from a chemical structure and biosynthesis perspective into fatty acids (FAs), glycerolipids, glycerophospholipids, sphingolipids (SPs), sterolipids, prenolipids, saccharolipids, polyketides, and others. Lipids play a crucial role in regulating various life processes, including energy conversion, substance transport, information recognition and transmission, cell development and differentiation, and apoptosis. Moreover, abnormal lipid metabolism is closely associated with the onset and progression of various diseases such as atherosclerosis, diabetes, obesity, Alzheimer's disease, and cancer (Yoon et al., 2022).

Compared to normal sperm motility values, patients with asthenozoospermia show a significant increase in the ratio of cholesterol sulfate to lipids in seminal plasma (Lopalco et al., 2019). Alcohol abuse impacts the lipid composition of seminal plasma, primarily manifested by a significant decrease in levels of sphingomyelin (SM) and phosphatidylethanolamine (PE), along with an increased cholesterol-to-phospholipid ratio (Gomathi et al., 1993). High-performance liquid chromatography–mass spectrometry analysis of human seminal plasma identified the three most abundant lipid classes as cholesterol, SM, and phosphatidylcholine (PC), while the three most varied classes were PC, PE, and triglyceride (TG) (Qi et al., 2018). In our analysis of lipids in seminal plasma from two groups, the top three categories in terms of abundance were PE, PC, and ceramide (Cer). Our lipid species detection was more comprehensive than that of Qi et al., but the top two categories remained the same. A study on oligozoospermia patients’ seminal plasma identified 37 metabolites with decreased concentrations, including 17 PCs and four SMs, with acylcarnitine and free l-carnitine being the most distinctive metabolites (Boguenet et al., 2020).

Our study revealed a decrease in TG content in the seminal plasma of necrozoospermia patients. As a primary energy source for many tissues, the identified differential lipids TG(4:0_14:1_16:0) and TG(4:0_14:1_18:0) suggest poor sperm vitality in necrozoospermia patients, correlated with low TG content in seminal plasma. This finding is consistent with the results of Li et al. indicating a correlation between decreased sperm vitality and low TG concentration in seminal plasma (Torres-Puig et al., 2022). Kim et al. demonstrated that local metabolism of TG in the male reproductive system can affect sperm production and vitality (Kim et al., 2017). A medium-chain TG-replacement diet can improve sperm quality in patients with asthenozoospermia, with low levels of serum non-esterified fatty acids being an effective non-pharmacological treatment method (Masaki et al., 2017). In azoospermia patients, the concentration of TG(14:1_16:0_18:3) in seminal plasma is one of the indicators predicting the success rate of sperm retrieval in testicular microdissection, with a sensitivity of 61%, specificity of 83%, and an AUC of 0.75 (Gamidov et al., 2022). Our study, using random forest model analysis, identified TG(4:0_14:1_16:0) as a potential biomarker for diagnosing necrozoospermia, with a sensitivity of 63%, specificity of 100%, and an AUC of 0.82.

It is demonstrated that PE(18:1e_22:4) and PE(18:0p_22:4) are downregulated among differential lipids. Yolk freezing protection solutions containing PE can reduce damage to spermatozoa membranes and vitality caused by freezing (Pillet et al., 2012). Lipidomic studies of seminal plasma in patients with pituitary stalk interruption syndrome observed a downregulation of PE, consistent with our research findings (Guo et al., 2022). Li et al. demonstrated that PE and TG serve as biomarkers for diagnosing Kallmann syndrome (Li et al., 2021). Phospholipids on the mitochondrial membrane primarily consist of phosphatidylcholine (PC), phosphatidylethanolamine (PE), and cardiolipin (CL), accounting for approximately 40%, 30%, and 15% of the total lipid composition, respectively (Samuel & Shulman, 2018). PE exhibits excellent antioxidant properties and plays a crucial role in semen liquefaction, inflammatory reactions, and semen coagulation (Deepinder et al., 2007). Mitochondria provide energy for most cells, and when the PE content in mitochondria decreases, cellular oxygen consumption, cellular adenosine triphosphate (ATP) levels, and the ATP generation rates significantly decrease. This indicates a correlation between the occurrence of necrozoospermia and a decline in spermatozoa mitochondrial function. A study by Somashekar et al. confirmed the crucial role of PE binding protein-4 (PEBP4) in spermatozoa production, maturation, and vitality (Somashekar et al., 2017). Its presence is positively correlated with regulating spermatozoa maturation and fertilization capability, suggesting PEBP4 as a potential reproductive indicator for spermatozoa quality and fertilization capability.

From the differential analysis, we selected lipids with a p-value ranking in the top 100 and identified lipid biomarkers through machine learning analysis using random forests. The top 5 important lipid features were LPE(20:4), SM(d36:2), MGMG(32:1), SM(t35:2), and TG(4:0_14:1_16:0). Lysophosphatidylethanolamine (LPE) is a product of phosphatidylethanolamine hydrolysis by phospholipase A1/A2 and is a component of cell membranes; it is identified as a potential biomarker for necrozoospermia, with a sensitivity of 75%, specificity of 56%, and an AUC value of 0.81 in this study. While this indicates a significant association with sperm vitality, we recognize its insufficient specificity and the wide confidence interval. An UPLC-MS-based seminal plasma lipidomics study identified lysophosphatidylethanolamine (LPE) with a specific acyl chain length of 20:4, revealing an increased pattern in seminal plasma across various physiological states of sperm, including activation and the acrosome reaction (Cheng et al., 2023). The upregulation of LPE 20:4 is particularly noteworthy as it may reflect dynamic changes in sperm membrane composition and function, which are crucial for sperm motility and fertilization capacity. This underscores the role of LPE 20:4 as a potential biomarker in seminal plasma, highlighting the significance of LPE 20:4 in the complex chain of events leading to successful fertilization. Further research is needed to elucidate the biological mechanisms of LPE(20:4) in sperm function and to confirm its utility as a diagnostic marker for necrozoospermia. Another lysophosphatidylethanolamine (LPE) with a specific acyl chain length of O-16:1 also shows a significant association with male infertility (Correnti et al., 2023). Levels of LPE (O-16:1) were found to be increased in the seminal plasma of infertile men. The increase in LPE may reflect alterations in the composition and function of the sperm membrane, which are crucial for the sperm viability and fertilization potential. The prospect of utilizing LPE as a biomarker is an area that future studies will aim to investigate, encompassing laboratory validation and a detailed testing of its correlation with the physiological state of sperm.

As a major lipid component of cell membranes, SM constitutes 12.5% of the total membrane lipids in human spermatozoa (Flesch & Gadella, 2000). The hydrolysis of SM to generate ceramide (Cer) increases spermatozoa acquisition capability. It also facilitates a faster and more intense acrosome reaction, promoting spermatozoa–egg fusion and enhancing the reproductive capacity of male animals (Ahumada-Gutierrez et al., 2019). SM synthase 2, located in the testes and spermatozoa of male animals, exhibits reduced expression in spermatozoa from patients with asthenozoospermia, indicating a correlation between decreased SM production and reduced spermatozoa motility (Li et al., 2020). SM(d36:2) and SM(d18:1_18:1) are differentially expressed lipids, with their downregulation indicating a correlation between the occurrence of necrozoospermia and decreased levels of SM in seminal plasma. In patients with pituitary stalk interruption syndrome, seminal plasma lipids such as SM(d38:2), SM(d40:2), SM(d36:1), SM(d34:1), SM(d42:1), and SM(d42:3) were also downregulated, aligning with our findings and suggesting abnormal nutritional conditions in the spermatozoa survival microenvironment (Guo et al., 2022).

## Strengths and limitations

This study employed an untargeted lipidomics approach to investigate the changes in seminal plasma lipids in necrospermia patients, providing a comprehensive understanding of the lipidomic profile in these individuals. Additionally, through the application of random forest machine learning, potential biomarkers were identified.

The current diagnostic methods for necrozoospermia predominantly involve traditional semen analysis, which assesses sperm count, motility, and morphology, alongside staining techniques such as Eosin-Nigrosin, which distinguishes live from dead sperm cells based on their ability to exclude or take up certain dyes (WHO, 2021). Despite their widespread use in clinical settings, we have noted that these methods may fail to provide adequate information in certain circumstances, particularly in cases of idiopathic necrozoospermia. Our study investigates lipidomics analysis as a promising adjunctive diagnostic tool, with the goal of improving the accuracy and reliability of diagnosis.

However, the study has its limitations as these putative biomarkers were not validated in vitro. The future research plans include conducting in vitro experiments to verify the specificity and sensitivity of these biomarkers, as well as to assess their accuracy in disease diagnosis. Another key aspect of ongoing research will involve developing a refined random forest algorithm model based on the lipidomic data of necrospermia patients. The model will undergo further external multi-centre validation to evolve into a more potent omics and Artificial Intelligence-based diagnostic tool. Such advancements would aim to predict fertility outcomes and assist clinicians in making more precise assisted reproductive strategies.

## Conclusions

LPE(20:4) and TG(4:0_14:1_16:0), were identified as differential lipids for necrozoospermia. Seminal plasma lipidomic analysis could provide valuable biochemical information for the diagnosis of necrozoospermia, and its combination with conventional sperm analysis may improve the accuracy and reliability of the diagnosis.

### Supplementary Information

Below is the link to the electronic supplementary material.

Supplementary file1 (XLSX 2511 KB)
Supplementary Datasheet 1: List of Sample Identification and Quantification

Supplementary file2 (XLSX 14 KB)
Supplementary Datasheet 2: Quantitative identification results of the top five lipids ranked by Random Forest in all samples

Supplementary file3 (DOCX 21 KB)
Supplementary Materials and Methods

## Data Availability

The raw data presented in this study are openly available in the OMIX database (https://ngdc.cncb.ac.cn/omix/view/OMIX006226): accession No. OMIX006226.

## Abbreviations

AD: Alzheimer’s disease
ATP: Adenosine triphosphate
BisMePA: Bis-methylphosphatidic acid
CASA: Computer-assisted semen analysis
Cer: Ceramide
CL: Cardiolipin
Co: Coenzyme
dMePE: Dimethylphosphatidylethanolamine
FDR: False discovery rate
GM: Ganglioside
Hex1Cer: Monohexosylceramide
Hex2Cer: Dihexosylceramide
Hex3Cer: Trihexosylceramide
LC–MS: Liquid chromatography–mass spectrometry
LPE: Lysophosphatidylethanolamine
MePC: Methylphosphatidylcholine
MS: Mass spectrometry
OPLS-DA: Orthogonal partial least square discriminant analysis
PC: Phosphatidylcholine
PCA: Principal component analysis
PE: Phosphatidylethanolamine
PEBP4: Phosphatidylethanolamine binding protein-4
PG: Phosphatidylglycerol
PI: Phosphatidylinositol
PLS: Partial least square
PLS-DA: Partial least square discriminant analysis
PS: Phosphatidylserine
QA: Quality assurance
QC: Quality control
ROC: Receiver operating characteristics
ROC: Receiver operating characteristics
RSD: Relative standard deviation
RSD: Relative standard deviation
SHOP: SHapley Additive exPlanations
SM: Sphingomyelin
SPH: Sphingosine
StE: Stigmasterol ester
SZMCH: Shenzhen Maternity & Child Healthcare Hospital
TG: Triglyceride
TG: Triglyceride
VIP: Variable importance in projection
