# Supplementary material for: Lipidomics random forest algorithm of seminal plasma is a promising method for enhancing the diagnosis of necrozoospermia
Source: Metabolomics. 2024 May 21;20(3):57. doi: 10.1007/s11306-024-02118-x (PMC11108888; doi:10.1007/s11306-024-02118-x)
Supplement: Supplementary file 3 — Supplementary file3 (DOCX 21 KB) Supplementary Materials and Methods [file 11306_2024_2118_MOESM3_ESM.docx]

**Supplementary Materials and Methods**

***Instruments and reagents***

The following instruments and reagents were utilized in the study: automated semen quality analyzer (SAS-II, Beijing, China), liquid chromatograph (Vanquish, Thermo Fischer Scientific, Waltham, MA), mass spectrometer (Q Exactive, Thermo Fischer Scientific), centrifuge (H1650-W, Changsha Xiangzhi Centrifuge Instrument Co., Ltd, Changsha, Hunan, China), vacuum concentrator (5305, Eppendorf, Hamburg, Germany), filter membrane (0.2-μm PTFE, Jin Teng Ltd., Hong Kong), shaker (BE-2600), methanol (CAS 67-56-1, purity ≥99.0%, Thermo Fischer Scientific), chloroform (CAS 67-66-3, purity ≥99.5%, Wokai, Oakland, CA), isopropanol (CAS 67-63-0, purity ≥99.9%, Thermo Fischer Scientific), H_2_O (7732-18-5, Milli-Q, Burlington, MA), acetonitrile (CAS 75-05-8, purity ≥99.9%, Thermo Fischer Scientific), formic acid (CAS 64-18-6, purity ≥98%, TCI), ammonium formate (CAS 540-69-2, purity ≥99%, Sigma Aldrich, St Louis, MO), and isopropanol (CAS 67-63-0, purity ≥99.9%, Thermo Fischer Scientific).

***LC-MS analysis***

The chromatography conditions were as follows:

- CQUITY UPLC® BEH C18 1.7 µm (2.1 × 100 mm) column.
- Autosampler temperature set at 8℃.
- Flow rate of 0.25 mL/min.
- Column temperature at 50℃.
- Injection volume of 2 μL for gradient elution.
- Mobile phase: acetonitrile:water, 60:40 (0.1% formic acid+10 mM ammonium formate) (A2) - isopropanol:acetonitrile, 90:10 (0.1% formic acid+10 mM ammonium formate) (B2).

Gradient elution program: 0–5 min, 70%–57% A2; 5–5.1 min, 57%–50% A2; 5.1–14 min, 50%–30% A2; 14–14.1 min, 30% A2; 14.1–21 min, 30%–1% A2; 21–24 min, 1% A2; 24–24.1 min, 1%–70% A2; 24.1–28 min, 70% A2.

The MS conditions were as follows:

- Instrument using electrospray ionization.
- Positive and negative ionization modes.
- Positive ion spray voltage at 3.50 kV, negative ion spray voltage at 2.50 kV.
- Sheath gas at 30 arb, auxiliary gas at 10 arb.
- Capillary temperature at 325℃, full scan at resolution 35,000, scan range 150–2,000.
- high energy collision dissociation(HCD )for second-level fragmentation, collision voltage at 30 eV, and dynamic exclusion to remove unnecessary MS/MS information [9,10].
